# Adiponectin receptor agonists inhibit leptin induced pSTAT3 and *in vivo* pancreatic tumor growth

**DOI:** 10.18632/oncotarget.19905

**Published:** 2017-08-03

**Authors:** Fanuel Messaggio, Alisha M. Mendonsa, Jason Castellanos, Nagaraj S. Nagathihalli, Lee Gorden, Nipun B. Merchant, Michael N. VanSaun

**Affiliations:** ^1^ Division of Surgical Oncology, Department of Surgery, Sylvester Comprehensive Cancer Center, University of Miami Miller School of Medicine, Miami, Florida, USA; ^2^ Department of Cancer Biology, Vanderbilt University Medical Center, Nashville, Tennessee, USA; ^3^ Department of Surgery, Vanderbilt University Medical Center, Nashville, Tennessee, USA

**Keywords:** adiponectin, AdipoRon, pancreatic adenocarcinoma, adipokine, STAT3

## Abstract

Obesity is a significant risk factor for pancreatic cancer, harboring a chronic inflammatory condition characterized by dysregulation of the adipokines, leptin and adiponectin, that in turn alter oncogenic signaling pathways. We and others have shown that leptin promotes the proliferation and an invasive potential of pancreatic cancer cells through STAT3 mediated signaling. However, the role of adiponectin on the tumorigenicity of pancreatic cancer has not been elucidated. Adiponectin represents an important negative regulator of cytokines, which acts through two receptors, ADIPOR1 and ADIPOR2, to elicit pro-apoptotic, anti-inflammatory, and anti-angiogenic responses. We show that the level and expression of both adiponectin receptors are decreased in pancreatic tumors relative to normal pancreatic tissue. *In vitro* stimulation with adiponectin or a small molecule adiponectin receptor agonist, AdipoRon, increases apoptosis while inhibiting pancreatic cancer cell proliferation, colony formation, and anchorage independent growth. In addition, adiponectin receptor agonism inhibits leptin mediated STAT3 activation. *In vivo*, treatment of mice with AdipoRon inhibits orthotopic pancreatic tumor growth. These results demonstrate that adiponectin receptor activation is a key regulator of pancreatic cancer growth and AdipoRon provides a rational agent for the development of novel therapeutic strategies for pancreatic cancer.

## INTRODUCTION

Pancreatic cancer ranks as the third leading cause of cancer related deaths and is projected to be the second by the year 2020. Therefore, improving our understanding of the biology of pancreatic ductal adenocarcinoma (PDAC) is of paramount importance [1, 2]. Epidemiological studies have linked pancreatic cancer risk with multiple factors including age, smoking, alcohol consumption, chronic pancreatitis, and Type II diabetes [3, 4]. The increasing incidence of pancreatic cancer is also associated with a rising prevalence of obesity, a documented adverse health risk for diabetes and pancreatitis [5–8].

Adipokines are adipose derived cytokines that can affect many aspects of cancer; acting as inflammatory mediators, growth factors, and angiogenic factors [9, 10]. The influence of adipokines on the initiation and progression of pancreatic cancer remains largely unknown. The adipokines, leptin and adiponectin, generally act in an opposing manner; leptin contributes to a pro-tumorigenic phenotype while adiponectin acts in an anti-tumorigenic manner. Stimulation of cancer cells with leptin leads to increased aggressiveness and metastatic phenotypes [11–13]. Conversely, adiponectin has been shown to inhibit proliferation, migration and invasion of breast cancer, prostate cancer and hepatic stellate cells [14–17]. Signal transducer and activator of transcription 3 (STAT3) signaling appears to be a key regulator of adipokine mediated activity. Administration of leptin to pancreatic cancer cell lines activates STAT3 signaling [13], whereas adiponectin inhibits leptin induced phosphorylation of STAT3 in prostate, esophageal and hepatocellular carcinoma cells [18, 19].

Reports on adiponectin levels in pancreatic cancer patients have been conflicting, however, high serum levels of adiponectin have been correlated with a decreased risk for pancreatic cancer [20, 21]. Importantly, a downregulation of adiponectin receptors has been observed in hepatocellular carcinoma and colorectal cancer [22, 23]. Therefore gaining an understanding of the regulation of adiponectin may have the potential to provide insights into the initiation and maintenance of pancreatic cancer and provide opportunities for novel treatment options. We show that there is a reduction in adiponectin receptor levels in mouse and human pancreatic cancer tumors and cell lines relative to normal pancreas tissue and report that supplementation with adiponectin or the adiponectin receptor agonist, AdipoRon, suppresses leptin-induced STAT3 signaling *in vitro* and reduces PDAC tumor growth *in vivo*. Our results suggest that adiponectin receptor agonism represents a key regulatory mechanism for pancreatic cancer with novel therapeutic potential.

## RESULTS

### Adiponectin receptor levels are decreased in pancreatic tumors

**Figure 1:**
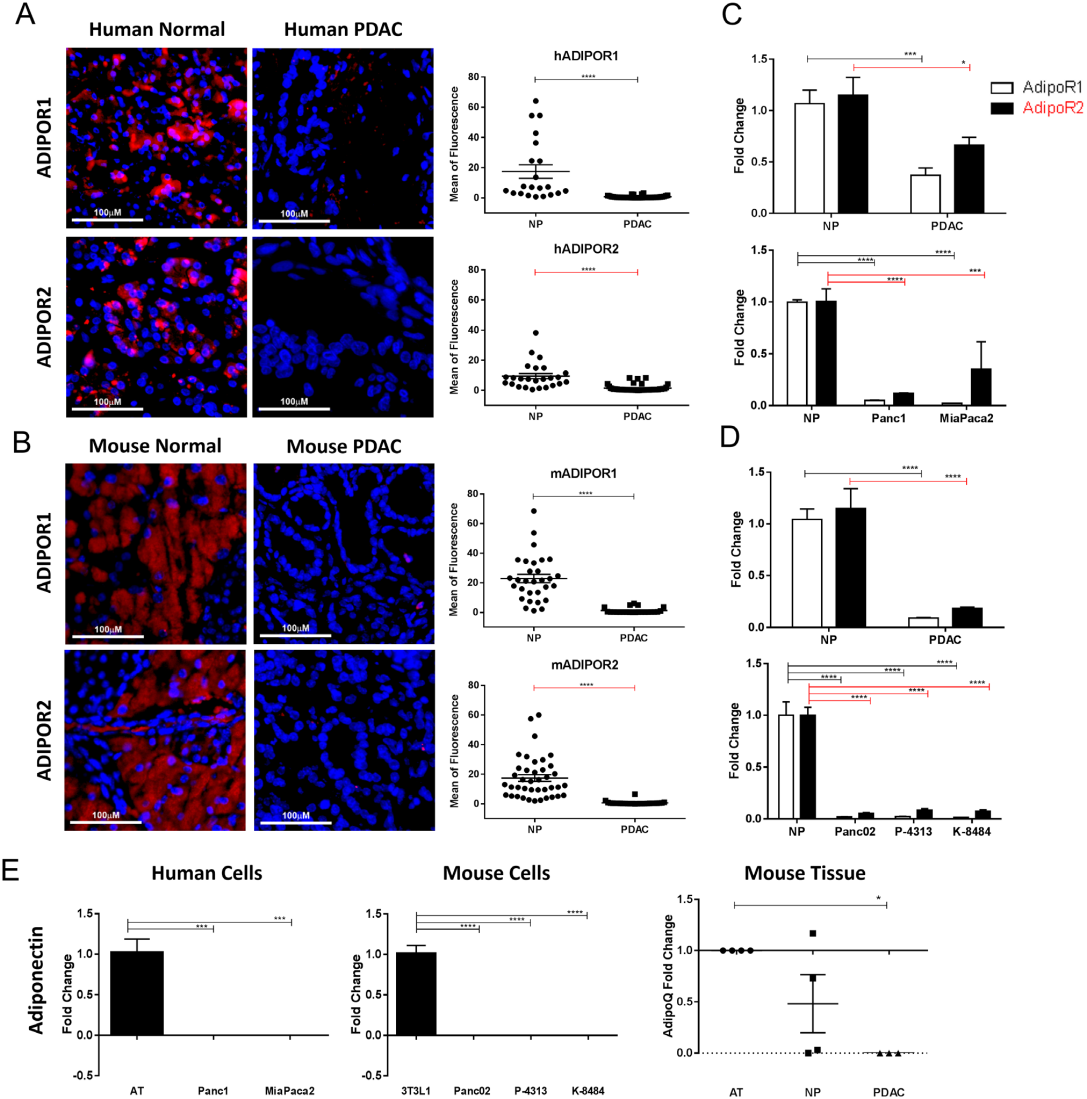
Adiponectin receptor levels are reduced in pancreatic cancer Human and murine normal and pancreatic cancer samples were immunostained for ADIPOR1 and ADIPOR2. **(A)** strong staining is present in the acinar tissue of human normal samples for both receptors but is decreased in the human pancreatic tumor samples. **(B)** murine pancreatic cancer tissue samples from PKT mice demonstrate a high level of staining in the adjacent acinar tissue and a reduced staining in the dysplastic pancreatic tumor. Graphs represent quantitative analysis of mean fluorescence from single channel images. Scale bar is 100μm. **(C-D)** quantitative real time PCR analysis was used to determine the relative expression of *AdipoR1* (white bars) and *AdipoR2* (black bars) in human and murine pancreatic tumor samples (PDAC) as well as in pancreatic cancer cell lines, relative to the level expressed in the normal pancreas (NP) tissue. **(E)** quantitative real time PCR analysis for adiponectin expression in human and mouse pancreatic cancer cell lines and mouse tissue, relative to human adipose tissue (AT), 3T3L1 cells differentiated to adipocytes (3T3L1) and mouse AT, respectively. Statistical analysis was performed separately for ADIPOR1 and ADIPOR2 protein levels (unpaired t-test) as well as *AdipoR1* and *AdipoR2* expression (two-way Anova), or adiponectin expression (ordinary one-way Anova) (*P ≤ 0.05, ***P ≤ 0.001, ****P ≤ 0.0001).

While the presence of both ADIPOR1 and ADIPOR2 has previously been reported in human PDAC tumor samples [24], the localization and the expression level of these receptors relative to normal tissue has not been investigated. Using a human tissue microarray of normal pancreas and PDAC samples, we identified ADIPOR1 and ADIPOR2 to be primarily localized to the normal acinar tissue. In human PDAC tumors, the level of both ADIPOR1 and ADIPOR2 receptors were significantly decreased relative to normal acinar tissue (Figure 1A). PDAC samples collected from a genetically engineered mouse (GEM), PKT (*Ptf1a*^*cre/+*^; *Kras*^*G12D/+*^*; Tgfbr2*^*fl/fl*^), also showed a decrease of both ADIPOR1 and ADIPOR2 in tumor tissue compared to the adjacent normal acinar tissue (Figure 1B). High power images of pancreatic tissue co-stained for peanut agglutinin (PNA) and ADIPOR1 or ADIPOR2 confirmed their localization with acinar tissue (Supplementary Figure 1A, 1B).

We further characterized the mRNA expression of *AdipoR1* and *AdipoR2* in human and murine pancreatic tumor tissue as well as PDAC cell lines relative to normal pancreas utilizing quantitative PCR analysis. Expression levels of *AdipoR1* and *AdipoR2* were decreased in human PDAC and in cell lines, Panc1 and MiaPaca2, compared to normal human pancreatic tissue (Figure 1C). Expression levels of *AdipoR1* and *AdipoR2* were also decreased in murine pancreatic cancer samples and in cell lines Panc02, P-4313, and K-8484 when compared to normal murine pancreatic tissue (Figure 1D). Adiponectin itself has not been reported to be expressed by pancreatic cancer cells at significant amounts. Therefore, we characterized the expression of adiponectin from cancer cells and pancreatic tissues using qRT-PCR. Pancreatic cancer cell lines did not express detectable levels of adiponectin in comparison to the robust expression in differentiated 3T3-L1 cells or peripheral adipose tissue (Figure 1E). We were able to detect expression of adiponectin in two of four samples of normal pancreas, yet expression was not detected from murine PDAC lysates (Figure 1E).

### Full length adiponectin inhibits proliferation of pancreatic cancer cells

**Figure 2:**
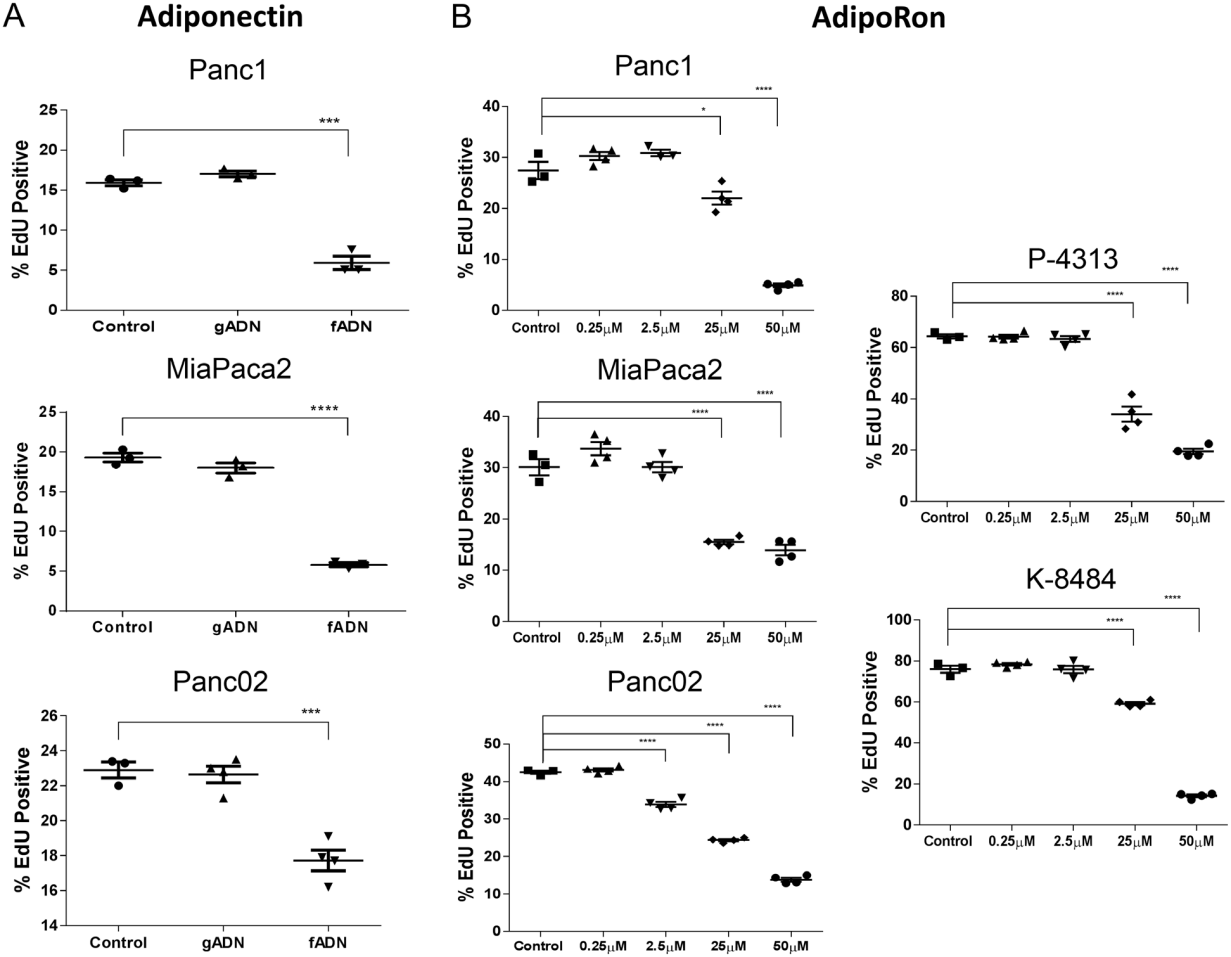
Adiponectin receptor agonists inhibit pancreatic cancer cell proliferation **(A)** recombinant globular adiponectin (gADN) at 1μg/mL or full length adiponectin (fADN) at 10μg/mL were incubated with human (Panc1 and MiaPaca2) and mouse (Panc02) pancreatic cancer cell lines, labeled with EdU and measured for active proliferation. DMSO was used as a control. Statistical analysis was performed using unpaired t-test (***P ≤ 0.001, ****P ≤ 0.0001). **(B)** human (Panc1 and MiaPaca2) and mouse PDAC cells (Panc02, P-4313 and K-8484) were treated with increasing concentration of AdipoRon for 48h before measuring proliferation via EdU, which showed dose-dependent inhibition for each PDAC cell line. Statistical significance was determined using ordinary one-way Anova (*P ≤ 0.05, ****P ≤ 0.0001).

Adiponectin is secreted as a full length monomeric protein that can either aggregate into multimeric high molecular weight (HMW) clusters or be cleaved to generate globular ligands [25, 26]. ADIPOR1 preferentially responds to the globular form of adiponectin while ADIPOR2 responds to the full length monomeric and HMW forms [27]. Total levels of adiponectin in lean mice averaged 8.77μg/mL (Supplementary Figure 3C). Of note, the globular form of adiponectin circulates at low abundance in human plasma as compared to full length adiponectin [28]. In order to determine the effect of adiponectin on the proliferation of pancreatic cancer cells, we exposed murine and human pancreatic cancer cells to either globular adiponectin, gADN (1μg/mL) or full-length recombinant adiponectin, fADN (10μg/mL). The globular form of adiponectin had no effect on cell proliferation, while the full length form of adiponectin significantly inhibited proliferation of the Panc1, MiaPaca2, and Panc02 cells (Figure 2A).

### AdipoRon, an adiponectin agonist, inhibits pancreatic cancer cell growth *in vitro*

**Figure 3:**
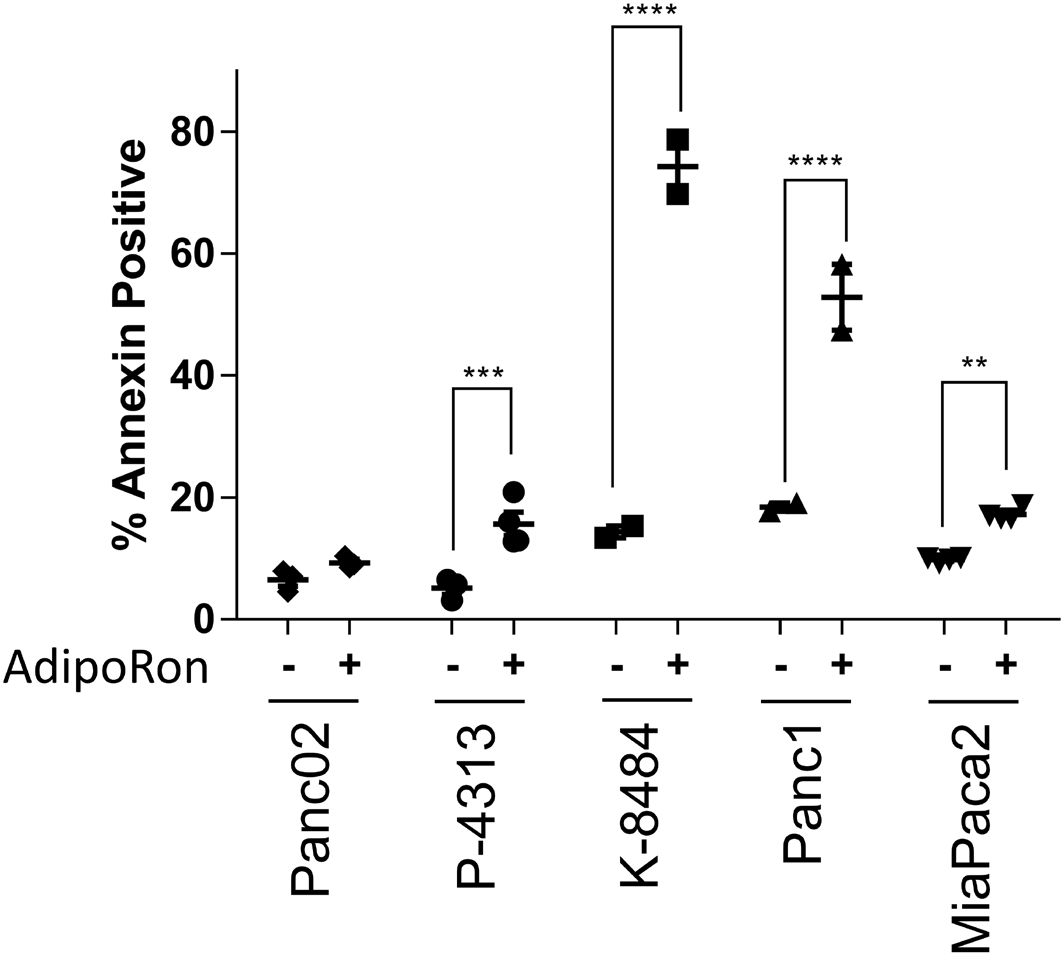
Adiponectin receptor agonist AdipoRon promotes pancreatic cancer cell apoptosis Annexin V staining was used to evaluate the change in percentage of apoptotic cells by comparing 50μM AdipoRon treatment (+) versus control (-) in multiple PDAC cell lines. Statistical significance was determined using ordinary one-way Anova test (**P ≤ 0.01, ***P ≤ 0.001, ****P ≤ 0.0001).

Recently, AdipoRon, a small molecule agonist of both adiponectin receptors ADIPOR1 and ADIPOR2, was shown to reduce diabetic symptoms in a genetically obese mouse model [29]. An effect not observed in double-knockout AdipoR1/AdipoR2 mice. In order to determine if AdipoRon could be used to treat PDAC, we administered AdipoRon to pancreatic cancer cells and assessed them for viability. The number of proliferating cells and the number of apoptotic cells were each quantified after AdipoRon treatment in mouse Panc02, P-4313 (obtained from GEM; Kras^G12D/+^, Pdx^1cre/+^) and K-8484 (obtained from GEM; Kras^G12D/+^, p53^R172H/+^, Pdx1^cre/+^) cell lines as well as human Panc1 and MiaPaca2 cell lines. AdipoRon administration to PDAC cells resulted in a significant, concentration-dependent reduction of cell proliferation (Figure 2B) as well as increased apoptosis (Figure 3).

**Figure 4:**
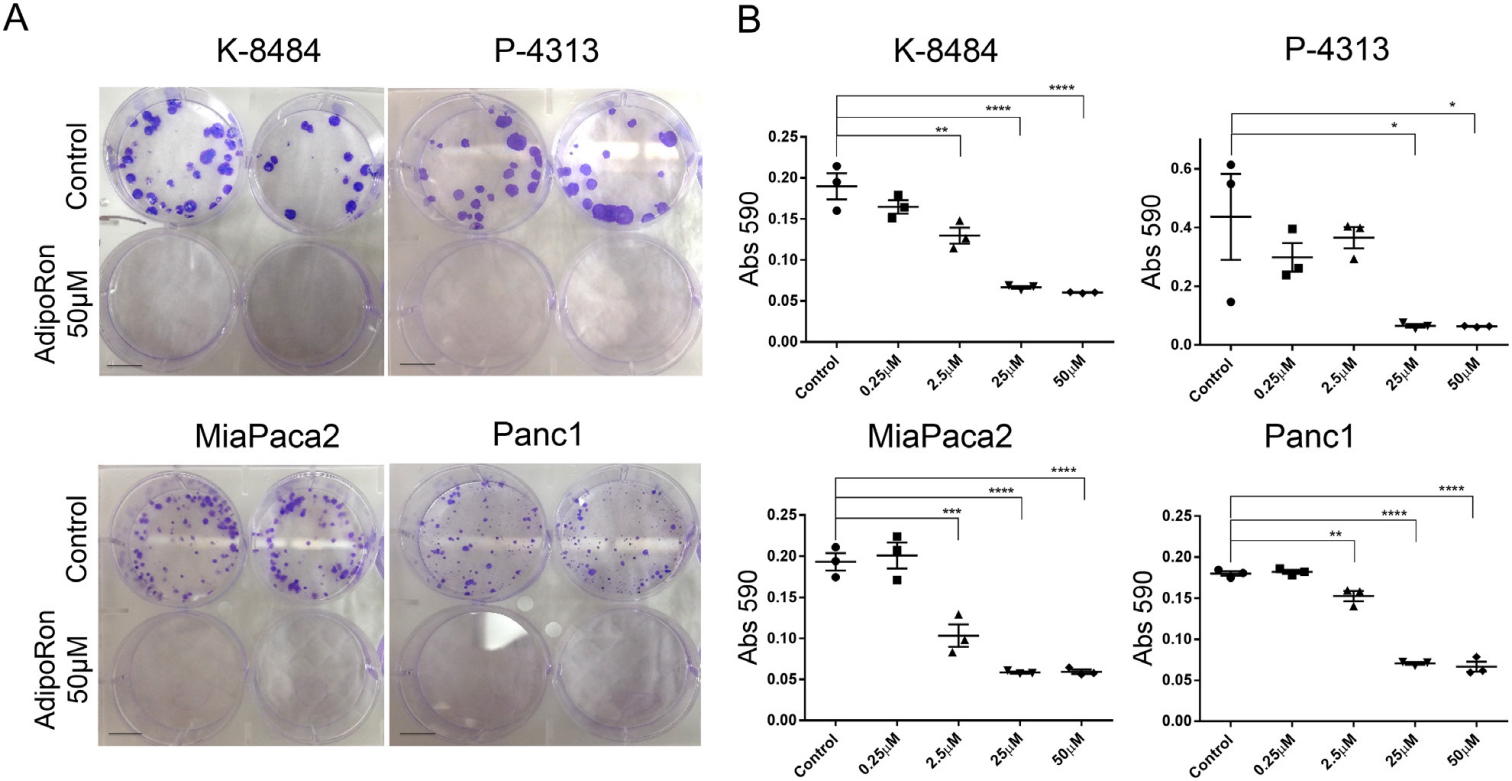
AdipoRon inhibits colony formation of pancreatic cancer cells PDAC cell lines were treated with an increasing concentration of AdipoRon for 2 weeks. **(A)** representative images show a drastic decrease in colony number and size after AdipoRon treatment compared to control. **(B)** crystal violet staining was performed and quantification of stain was determined by reading absorbance at 590nm. Colony formation was decreased for both murine and human pancreatic cancer cell lines. Statistical analysis was performed using ordinary one-way Anova test (*P ≤ 0.05, **P ≤ 0.01, ***P ≤ 0.001, ****P ≤ 0.0001).

**Figure 5:**
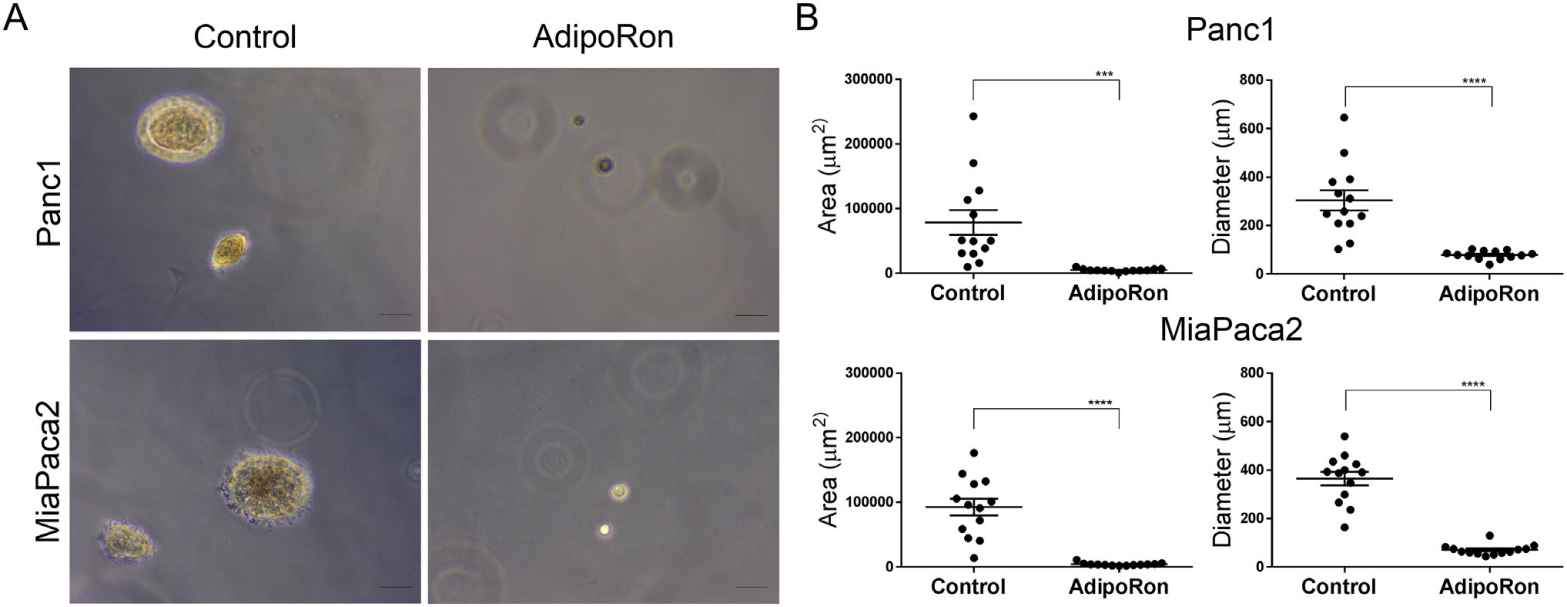
AdipoRon inhibits anchorage independent growth PDAC cell lines were seeded in soft agar and treated with an increasing concentration of AdipoRon for 2 weeks. **(A)** anchorage independent growth was evaluated in the presence or absence of 50μM AdipoRon. **(B)** quantitative assessment of spheroid area as well as spheroid diameter were both decreased with AdipoRon treatment compared to control. Statistical analysis was performed using ordinary one-way Anova (***P ≤ 0.001, ****P ≤ 0.0001).

To determine the effectiveness of prolonged exposure to AdipoRon, PDAC cells were plated at low density and treated with AdipoRon in a concentration dependent manner for 2 weeks, which resulted in an effective growth suppression of PDAC cell colonies when compared to DMSO control treated cells (Figure 4A, 4B). Furthermore, AdipoRon treatment caused a significant decrease in anchorage independent growth as measured by overall spheroid area and diameter of Panc1 and MiaPaca2 cells (Figure 5A, 5B). Collectively, these results demonstrate that the adiponectin receptor agonist, AdipoRon, inhibits PDAC tumorigenesis *in vitro*.

### AdipoRon inhibits leptin mediated STAT3 activation

**Figure 6:**
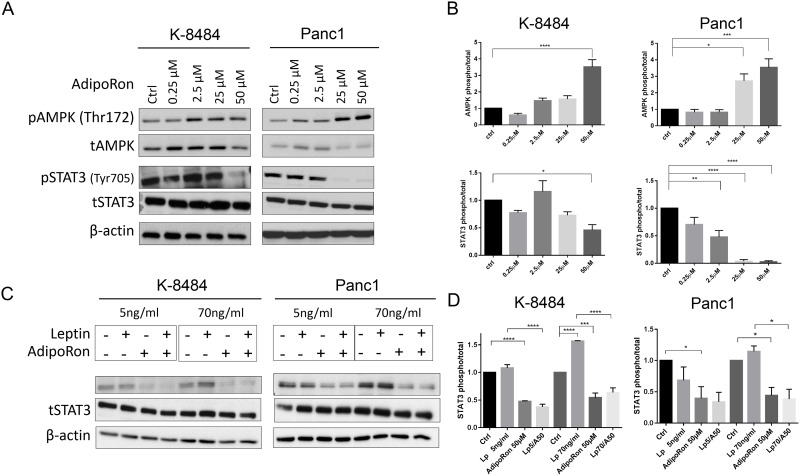
AdipoRon inhibits cytokine-induced pSTAT3 **(A)** qualitative representation of mouse K-8484 and human Panc1 PDAC cells starved overnight and then treated with increasing concentrations of AdipoRon for 12h. At the endpoint cells were lysed and probed with either pAMPK, tAMPK, pSTAT3, or tSTAT3 antibody. **(B)** correspondent relative quantitation of triplicate experiments. **(C)** AdipoRon inhibits Leptin-induced STAT3 activation. K-8484 and Panc1 PDAC cell lines were starved overnight and then treated with Leptin at lean concentration of 5ng/ml or an obese concentration of 70ng/ml alone or in combination with 50μM AdipoRon for 12h. Western analysis demonstrated suppression of leptin-induced pSTAT3 in the presence of AdipoRon. **(D)** correspondent relative quantitation of triplicate experiments. β-actin levels are shown as a total loading control. Statistical analysis was performed using ordinary one-way Anova (*P ≤ 0.05, **P ≤ 0.01***P ≤ 0.001, ****P ≤ 0.0001).

Upon receptor binding, adiponectin classically activates AMPK signaling through the APPL1 adaptor protein [30]. To determine the molecular mechanism of AdipoRon in PDAC cells, murine K-8484 and human Panc1 cells were treated with increasing concentrations of AdipoRon. Similar to adiponectin, AdipoRon activated pAMPK signaling (Figure 6A, 6B) and subsequently increased pACC (Supplementary Figure 2). Additionally, AdipoRon treatment of PDAC cells resulted in a decreased level of pSTAT3. We have previously shown that leptin activates STAT3 phosphorylation in PDAC cells [13]. We now show that AdipoRon inhibits leptin induced pSTAT3 activation in PDAC cells at both lean (5ng/mL) and obese (70ng/mL) concentrations of leptin (Figure 6C, 6D). These results confirm that during concurrent activation, adiponectin signaling counters the mitogenic effects of leptin in PDAC cells.

### AdipoRon inhibits pancreatic tumor growth *in vivo*

**Figure 7:**
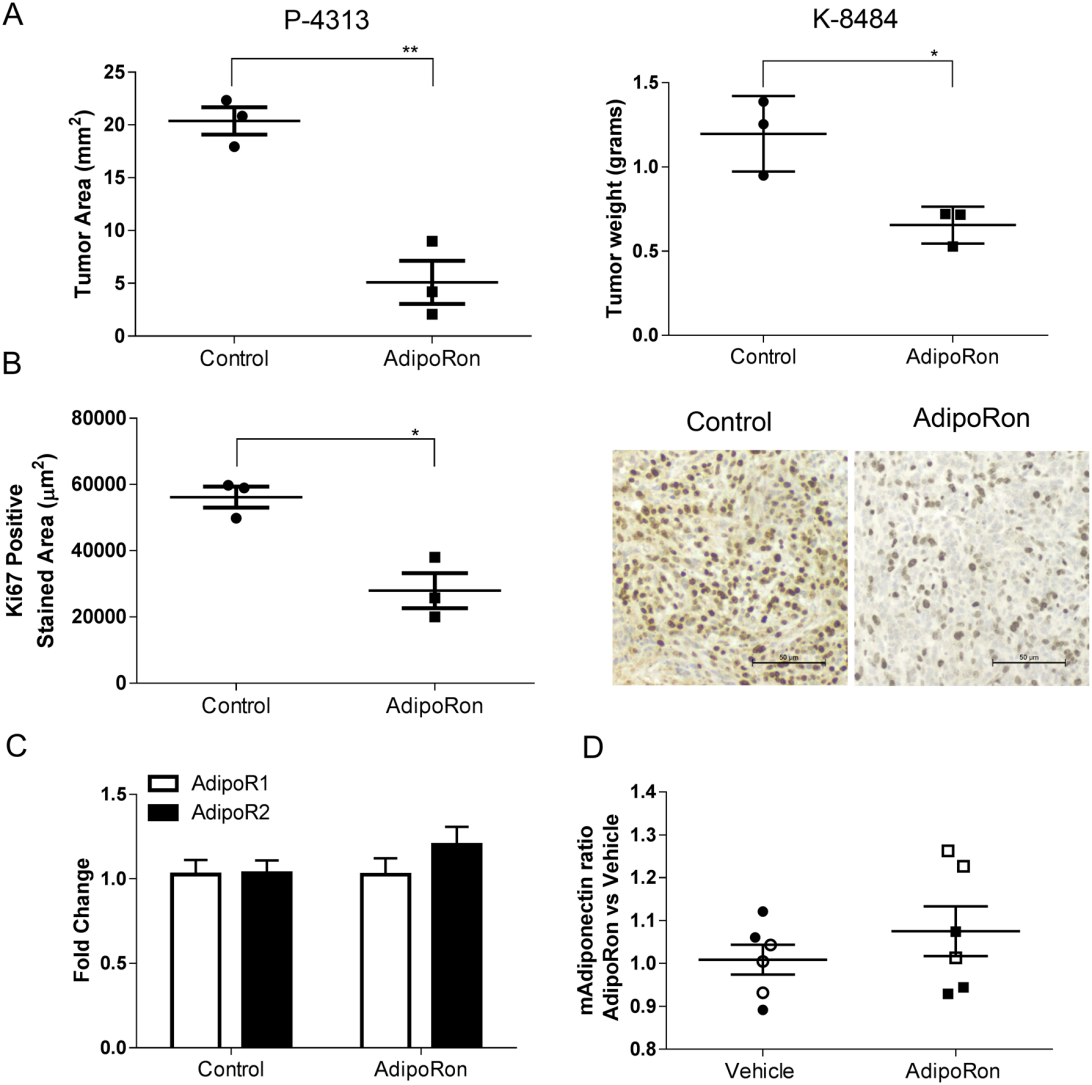
AdipoRon inhibits orthotopic pancreatic tumor growth and tumor proliferation *in vivo* Murine P-4313 and K-8484 cancer cells were orthotopically injected into syngeneic wildtype mice and allowed to grow for two weeks, after which mice were treated with vehicle or AdipoRon 5mg/kg/day for 2 weeks. **(A)** at endpoint, AdipoRon treated mice had a significantly reduced tumor size. **(B)** tumor proliferation measured by Ki67 positive tumor area per tumor section was significantly reduced after AdipoRon treatment. **(C)** no significant difference was detected in relative expression of AdipoR1 (white bars) and AdipoR2 (black bars) by quantitative real time PCR analysis between control and AdipoRon treated tumors. **(D)** ELISA analysis showed no significant difference in circulating serum adiponectin levels between the two groups of mice. (P-4313 filled symbols and K-8484 open symbols). Statistical analysis was performed using unpaired t-test (*P ≤ 0.05, **P ≤ 0.01).

We then sought to determine the effects of AdipoRon on pancreatic tumor growth *in vivo*. Murine P-4313 Kras^G12D^ mutant or K-8484 Kras^G12D^/p53^R172H^ pancreatic cells were injected into the pancreas of naïve mice and allowed to establish for two weeks before treating the mice with either vehicle or AdipoRon (5mg/kg/day). AdipoRon treated mice showed a significant reduction in the growth of orthotopic pancreatic tumors compared to vehicle treated tumors (Figure 7A). The level of tumor proliferation, as measured by Ki67 positive cells per tumor area, was significantly reduced in AdipoRon treated mice compared to vehicle treatment (Figure 7B). In a comparative model, syngeneic Panc02 (derived from a carcinogen induced tumor) or P-4313 pancreatic cancer cells were injected orthotopically into adiponectin deficient mice. The absence of adiponectin resulted in an increased growth of Panc02 tumors, yet did not significantly affect the growth of P-4313 tumors when compared to respective tumors in wild-type mice (Supplementary Figure 3). These results further validate the role of adiponectin in inhibiting pancreatic tumor growth. ELISA based analysis of serum from adiponectin deficient mice (Supplementary Figure 3C) or conditioned media from murine PDAC cell lines (Supplementary Figure 3D) revealed undetectable levels of adiponectin in comparison to wildtype mice or differentiated 3T3-L1 adipocytes respectively.

## DISCUSSION

Obesity is commonly associated with multiple forms of cancer including breast, colon, pancreas, prostate and uterine [31]. Serum levels of adipokines change greatly in obese individuals, resulting in decreased levels of adiponectin and increased levels of leptin [32–34]. The adipokines leptin and adiponectin are known to affect multiple aspects of cancer progression [35]. The overall objective of these studies was to understand pancreatic cancer growth in response to adiponectin or a small molecule adiponectin receptor agonist, AdipoRon. In this study we provide the first evidence that both adiponectin and AdipoRon inhibit pancreatic tumor growth and overcome leptin mediated mitogenic signaling.

Immunohistochemical analysis of human and murine PDAC tumors showed a downregulation of both adiponectin receptors, ADIPOR1 and ADIPOR2, in cancer compared to adjacent normal acinar tissue. In comparison, adiponectin receptors levels have also been shown to be significantly lower in human hepatocellular carcinoma [22], high-grade endometrial adenocarcinoma [36], and in colorectal cancer [37] when compared to adjacent non-neoplastic tissue. Furthermore, a lower expression of these receptors correlated with an increased risk of recurrence in hepatocellular carcinoma and poorly differentiated colorectal cancers [23].

To date, no studies have identified a mechanism for the regulation of adiponectin receptors in pancreatic cancer. Mutations in the adiponectin receptor have been reported, yet they are infrequent and it is likely that epigenetic changes in the adiponectin receptor promoter could be an important factor in PDAC. Obesity has been demonstrated to cause hypermethylation of the adiponectin [38] leading to subsequent suppression of adiponectin expression. Epigenetic analysis for adiponectin, AdipoR1, and AdipoR2 in PDAC will help to clarify the molecular mechanisms regulating expression in PDAC. We have found that although adiponectin receptors are expressed at low levels in PDAC, it is either sufficient enough to respond to increased levels of adiponectin/AdipoRon or alternate receptors such as T-Cadherin may be compensating.

Previous studies have shown that adiponectin can inhibit the growth of a variety of cancer cells in culture [15, 17, 39–41]. We show that stimulation with full length adiponectin inhibited proliferation of pancreatic cancer cells *in vitro* at 10μg/mL, a concentration in the range of circulating blood levels in healthy patients. Furthermore, the small molecule agonist AdipoRon was also effective at inhibiting proliferation as well as inducing apoptosis in a panel of murine and human pancreatic cancer cell lines. AdipoRon may represent an economical pharmacological method to achieve super-physiological levels of adiponectin as a receptor agonist.

Recently, two reports preliminarily addressed pancreatic cancer growth in adiponectin deficient mice using variants of the murine Panc02 pancreatic cancer cell line with opposing results [42, 43]. Kato, et al. showed that loss of adiponectin resulted in increased tumor size of orthotopically implanted PDAC tumors and decreased apoptosis [42]. In contrast, Huang, et al., showed decreased tumor growth and increased cleaved caspase-3 levels in flank injected PDAC tumors grown in adiponectin deficient mice [43]. The discrepancy of these results might be explained by microenvironmental factors related to the location of the implanted tumor. Similar to the Kato study, we found an increased growth of Panc02 tumors orthotopically implanted into adiponectin deficient mice. Interestingly, P-4313 cells did not demonstrate a growth benefit from loss of adiponectin. One possible difference in growth could be due to presence of mutant Kras in the P-4313 cells, as the Panc02 do not have a documented Kras mutation. Yet, determining whether mutant Kras is responsible for this growth effect would require additional validation. Importantly, neither study tested whether adiponectin treatment could be used to inhibit pancreatic tumors *in vivo*. Our studies demonstrate that AdipoRon significantly decreases the size and proliferation of P-4313 and K-8484 orthotopic pancreatic tumors *in vivo*.

Activation of STAT3 has been shown to occur frequently in cell lines and in human pancreatic cancers, which correlates with progression of disease [44–47]. Our previous studies showed that leptin induced phosphorylation of STAT3 in PDAC cells [13]. Our group has also shown that activated STAT3 signaling is a key biomarker of resistance and confers a poor outcome in PDAC patients [44]. Here we show that adiponectin suppresses leptin induced STAT3 signaling in PDAC. Leptin induced STAT3 phosphorylation has also been shown to be inhibited by addition of adiponectin in hepatocellular cancer cells, which is associated with increased suppressor of cytokine signaling 3 (SOCS3) signaling, a negative regulator of STAT3 [41]. Inhibition of STAT3 signaling via adiponectin receptor activation provides a novel approach to inhibit PDAC tumor growth and progression and perhaps enhance therapeutic response to cytotoxic chemotherapy.

In conclusion, we show that the two main receptors for adiponectin, ADIPOR1 and ADIPOR2, are downregulated in PDAC relative to normal pancreas. Restoration of healthy levels of adiponectin or supplementation with AdipoRon, even when adiponectin receptor expression is low, results in a generalized inhibition of cell proliferation of PDAC both *in vitro* and *in vivo*. We believe that reduction of adiponectin in obese individuals reduces the counteraction of stimulatory cytokines, such as leptin leading to a more severe phenotype. Our results are the first to demonstrate that the adiponectin receptor agonist AdipoRon inhibits pancreatic tumor growth and proliferation, thereby supporting its role as a potential therapeutic agent in pancreatic cancer.

## MATERIALS AND METHODS

### Cell lines and culture

MiaPaca2 and Panc1 cell lines were purchased directly from ATCC and passaged less than six months. The Panc02 cell line was acquired from the NIH repository. The following cell lines were kindly donated: P-4313 (Kras^G12D/+^) cell line was obtained from Dr. Lowy (University of California, San Diego) (32); K-8484 (Kras^G12D/+^; p53^R172H/+^) cell line from Dr. Tuveson (Cambridge Research Institute, Cancer Research UK) (30). Cell lines are routinely authenticated by microscopic analysis and all tested clean of mycoplasma. Human cells were authenticated via STR profiling from IDEXX BioResearch (Westbrook, Maine 04092, USA) and mouse cell lines were verified for expression of KRasG12D and lack of αSMA. MiaPaca2, Panc1, Panc02 and K-8484 cell lines were maintained *in vitro* with of 10% fetal bovine serum (Omega Scientific Inc., FB-12) and 1x anti-anti (Gibco, 15240-062) in Dulbecco’s Modified Eagle Media (Corning Cellgro, 10-014-CV). The P-4313 cell line was maintained in RPMI Medium 1640 (Gibco, A10491-01) supplemented with 10% FBS, 1x Anti-anti, 1x Vitamins (Gibco, 11120-052) and 1x NEAA (Gibco, 11140-050). All cells were maintained at 37°C and 5% CO_2_. AdipoRon was purchased from Enzo Life Science (ENZ-CHM101). Recombinant globular adiponectin (1688-AC or 1119-AC), full length adiponectin (1065-AP or 5095-AC), and Leptin (Mouse 498-OB or human 398-LP) from R&D System was used for *in vitro* analysis.

### Animal studies

All experimental procedures using laboratory animals were approved by the animal care committee of Vanderbilt University and University of Miami. Wildtype (000664) and Adiponectin deficient (008195) mice were obtained from Jackson laboratories. Orthotopic pancreatic tumors were generated by surgically isolating the pancreas and implanting tumor cells directly into the tail of the pancreas in two month old mice. Pancreatic cancer cell lines P-4313 and K-8484 cells were trypsinized from cultures and counted on a Bio-Rad TC20. 2.5 million cells were resuspended in pharmaceutical grade phosphate buffered saline. 30μl of cell suspension was injected into the tail of the pancreas using a 27 gauge syringe. For AdipoRon treatment studies, P-4313 tumors and K-8484 were allowed to grow for 14 days and then animals were administered vehicle or AdipoRon (5mg/kg/day) for two weeks. At the end of each study, mice body weight was measured and then the pancreas was removed, weighed, and processed for histological and molecular analysis.

### Immunofluorescence

Murine pancreatic cancer tissues were obtained from 4-6 week old PKT (Ptf1a^cre/+^; Kras^G12D/+^; Tgfbr2^fl/fl^) mice and normal pancreas tissue was obtained from littermate mice control, not expressing Cre. Both male and female mice were used for these studies. Pancreas tissue was resected, fixed in buffered formalin overnight, and then paraffin embedded. De-identified human normal tissue samples were obtained from Vanderbilt Translational Pathology Shared Resource. Tissue microarrays of human normal and pancreatic cancer samples were obtained from BioMax (PA483c). Antigen retrieval was performed with Proteinase K (for ADIPOR2) or Citric Buffer (for ADIPOR1). Sections were washed and then endogenous peroxidases quenched with 3% peroxide in TBS before blocking (5% normal donkey serum, 1% BSA, 0.1M MgCl2, 0.5% Tween 20, and 10mM Tris pH 7.4). Sections were stained with antibodies to ADIPOR1 (ThermoFisher Scientific, PA1-059), ADIPOR2 (ThermoFisher Scientific, PA1-1071) overnight at 4°C. For detection, sections were labeled with appropriate species specific secondary antibody Alexa Fluor 594 and Alexa Fluor 488 (Life Technologies). Slides for co-staining were incubated for 30min at room temperature in Rhodamine Peanut Agglutinin (PNA; Vector labs RL-1072). Slides were additionally counterstained with Dapi or NucBlue Fixed Cell Stain (Molecular Probes, R37606) and mounted with Permount (Fisher Scientific, SP15-100). Slides were then scanned on a Zeiss Apotome at the Sylvester Cancer Center Analytic Imaging Core Facility. Multiple fields for multiple samples were acquired and normalized to background IgG levels. Specifically, 5 normal mouse pancreatic tissue, 5 mouse pancreatic adenocarcinoma tissue samples and 3 normal pancreatic tissue samples were evaluated, 6 field per sample were acquired and analyzed. One field per sample was acquired and analyzed on TMA with 8 human normal pancreas together with 40 human pancreatic adenocarcinoma samples. Single channel grey scale images were analyzed with ImageJ software to evaluate the mean of fluorescence as a result of integrated fluorescence intensity per area field.

### Immunohistochemistry

Vehicle or AdipoRon treated orthotopic P-4313 and K-8484 pancreatic tumors were resected from C57bl/6J mice, fixed in buffered formalin overnight, and then paraffin embedded. 6μm sections were dewaxed and rehydrated through a decreasing percentage of ethanols. Heat-induced antigen retrieval for Ki67 was performed in 10mM sodium citrate buffer pH 6.0. Sections were washed and then endogenous peroxidases quenched with 3% peroxide in TBS before blocking (5% normal donkey serum, 1% BSA, 0.1M MgCl, 0.5% Tween 20, and 10mM Tris pH 7.4). Sections were stained with antibodies to Ki-67 (ab15580) overnight at 4°C. For detection, sections were labeled with appropriate species specific biotinylated secondary antibody (Vector Labs, Burlingane, CA), processed with a Vectastain kit (Vector Labs) and developed in chromogen solution (0.1 M Tris-HCl pH 7.4, 1.125 mM diaminobenzidine, 0.01% H2O2), counterstained with Mayer’s Hematoxylin Solution (Sigma), dehydrated with graded ethanols and mounted with Permount. Slides were then scanned on a Leica SCN400 Slide Scanner at the Vanderbilt Digital Histology Shared Resource. Ki67 was measured by averaging the level of staining per area of tumor from three different sections of each tumor. A total of three tumors for each treatment group were then analyzed using GraphPad Prism6. Pathological examination and staining assessment of tissue sections were verified microscopically.

### Reverse transcription and quantitative real-time PCR (qRT-PCR)

Total RNA was isolated from cultured cells and tissue samples using Qiazol (Qiagen, 79306) and chloroform extraction. The aqueous layer was then purified using RNeasy kit (Qiagen, 74104). The cDNA was generated from 1μg of total RNA via reverse transcription using High Capacity cDNA kit (Applied Biosystems, 4368814). Real-time PCR analysis was carried out following the iQ SYBR green supermix (Bio-Rad, 170-8882) on a CFX96 real time PCR detection system (Bio-Rad) using Qiagen QuantiTect transcript specific primers for mouse or human ADIPOR1 (QT00154217, QT00002352), ADIPOR2 (QT00165326, QT00058716) and ADIPOQ (QT01048047, QT00014091). Each sample was run in triplicate and fold-change was evaluated relative to normal samples and determined using GAPDH levels as a reference.

### Western blot analysis

For the assessment of STAT3, AMPK, or ACC level and phosphorylation status, total cell protein extracts were obtained from human and mouse PDAC cell lines, as well as from mouse tissue samples, via lysis and sonication in RIPA buffer (Cell Signaling, 9806). Total cell lysates were resolved by a 7.5% SDS-PAGE and probed with phospho-STAT3 (Cell Signaling, 9145S), phospho-AMPK (Cell Signaling, 2535S), phospho-ACC (Cell Signaling, 11818T), total STAT3 (Cell Signaling, 9139S) total AMPK (Cell Signaling, 5832S), and total ACC (Cell Signaling, 3676T). To determine equal loading, control membranes were probed with β-actin (Abgent, AM1829B). Quantitation of protein relative amounts of triplicate experiments were analyzed by ImageJ software as a ratio of each phospho protein band relative to the correspondent lane total protein band, followed by ratio to the lane’s loading control.

### EdU incorporation assay

Cells were dissociated with trypsin and counted on a Bio-Rad TC-20 and 5-10x10^5^ cells were seeded in each well of a 24 well plate and allowed to adhere in full media overnight. The next day, media was replaced with treatment media consisting of DMEM supplemented with 2.5% FBS and either DMSO, globular adiponectin (1μg/ml), full length adiponectin (10μg/mL), or AdipoRon (0.25-50μM) and allowed to grow for an additional 48h. EdU was then added to a final concentration of 10μM and incubated for 3 to 6 hours. Cells were lightly trypsinized and released with PEB (phosphate buffer pH 7.2, 2mM EDTA, and 1% BSA). Cells were filtered through 30μm filter and pelleted at 350xG. Cells were then fixed overnight at 4°C with 3% buffered formalin and subsequently permeabilized by addition of Triton (0.5%) for 10min. Cells were pelleted and washed with phosphate buffer and 1% BSA and then resuspended in labeling mix (150mM Tris pH 8.5, 1.5mM CuSO4, 2μM fluor-Azide dye). 100mM ascorbic acid was added to catalyze the reaction for 20min in the dark. A five-fold excess of PEB was added and the cells were pelleted at 350xG. Cells were then labeled with propidium iodide (0.5μg/mL) and assessed using a flow cytometer (CytoFLEX Flow Cytometer, Beckman Coulter, Inc.). Flow cytometry was performed with gating by side and forward scatter, eliminating cell doublets, then gating for propidium iodide positive cells. The percentage of EdU positive cells was gated from the total number of propidium iodide positive cells.

### Cell apoptosis assay

Cells were dissociated with trypsin, counted, and 100,000 Cells were seeded in each well of a 24 well plate and allowed to adhere in full media overnight. The next day, media was replaced with treatment media consisting of DMEM supplemented with 2.5% FBS and either DMSO or AdipoRon (50μM) and incubated for an additional 24h. Annexin V staining was performed following the manufacturer’s protocol (Life Technologies, V13241). Cells were assessed by flow cytometry comparing propidium iodide versus Annexin V positive cells.

### Colony formation assay

Pancreatic cancer cell lines were plated in triplicate for each treatment at a density of 5000 cells per well in a 6 well plate and allowed to adhere in complete media overnight. The day after, media was replaced with treatment media consisting of DMEM supplemented with 2.5% FBS and either DMSO or AdipoRon (0.25- 50μM). The incubation time for colony formation was 2 weeks for all cell lines following previously established protocols [48]. At the end of the treatment the media was gently removed from each of the wells by aspiration and cellular staining was performed for 20 minutes at room temperature with a solution of crystal violet (0.05% W/v crystal violet, 1% Formaldehyde, 1x PBS, 1% Methanol in dH_2_O). The excess crystal violet was washed with dH_2_O and the plates were allowed to dry at room temperature. Qualitative digital images of the colonies were scanned and analyzed. Elution of the crystal violet was performed using a 10% acetic acid solution for 15 minutes at room temperature. Eluates were diluted 1:4 in dH2O and the absorbance was read, in triplicate, at 590nm.

### Soft agar assay

A 1.6% Agar Solution (Sea Plaque Agarose, Lonza, 50101) was prepared in dH_2_O and autoclaved at 121°C for 15 minutes. The solution was kept at 56°C to avoid solidification. 2x RPMI solution was prepared by adding 10.3g of RPMI-1640 powder (Sigma, R7755) in 500ml of sterile dH_2_O, followed by the addition of 2.5% Fetal Bovine Serum, 1x L-Glutamine (Gibco, 25030-081) and 0.2% sodium bicarbonate (Gibco, 25080-094). For the bottom assay layer, 1.6% Agar Solution was mixed with the RPMI 2x solution at 1:1 ratio and 500μl per well was used to coat 24 well plates. Plates were then left at room temperature for 30 minutes to solidify. Pancreatic cancer cell lines were trypsinized and counted. 5000 cells per well were resuspended in 2x RPMI solution, 1.6% agar solution was added at 1:1 ratio, and then cells were plate, in triplicate for each treatment atop the previously established bottom layer. Plates were left at room temperature for 30 minutes to solidify. Cells were then incubated in a humidified 5% CO_2_ environment at 37°C for 2 weeks. At the endpoint, qualitative digital images of the colonies were taken and quantification for area and diameters of colonies for at least 15 fields were measured using ImageJ software.

### Mouse adiponectin enzyme-linked immunosorbent assay (ELISA)

The level of adiponectin from mouse cell line condition media or mouse serum samples (1:4000 dilution) was detected using a mouse Adiponectin/Arcp 30 DuoSet ELISA Kit (R&D System, DY1119) and analyzed using a FLUORstar OPTIMA microplate reader (BMG Labtech) according to the manufacturer’s protocols.

### Statistical analysis

Results are expressed as mean ± SEM. Group results were compared by One-Way Anova or Two-Way Anova with multiple comparison or unpaired t-test when appropriate. P value < 0.05 was considered significant.

## SUPPLEMENTARY MATERIALS FIGURES



## Abbreviations

ADIPOR1: Adiponectin receptor 1
ADIPOR2: Adiponectin receptor 2
ADN, ADIPOQ: Adiponectin
APPL1: Adapter protein with PH domain and PTB domain and Leucine zipper
AMPK: AMP-activated protein kinase
AKT: Ak strain transforming
MAPK: mitogen-activated protein kinase
STAT3: signal transducer and activator of transcription 3
EdU: 5-ethynyl-2'-deoxyuridine
