# Supplementary material for: Adiponectin receptor agonists inhibit leptin induced pSTAT3 and in vivo pancreatic tumor growth
Source: Oncotarget. 2017 Aug 3;8(49):85378–91. doi: 10.18632/oncotarget.19905 (PMC5689616; doi:10.18632/oncotarget.19905)
Supplement: Supplementary file 1 [file oncotarget-08-85378-s001.pdf]

Adiponectin receptor agonists inhibit leptin induced pSTAT3 and *in vivo* pancreatic tumor growth

SUPPLMENTARY MATERIALS

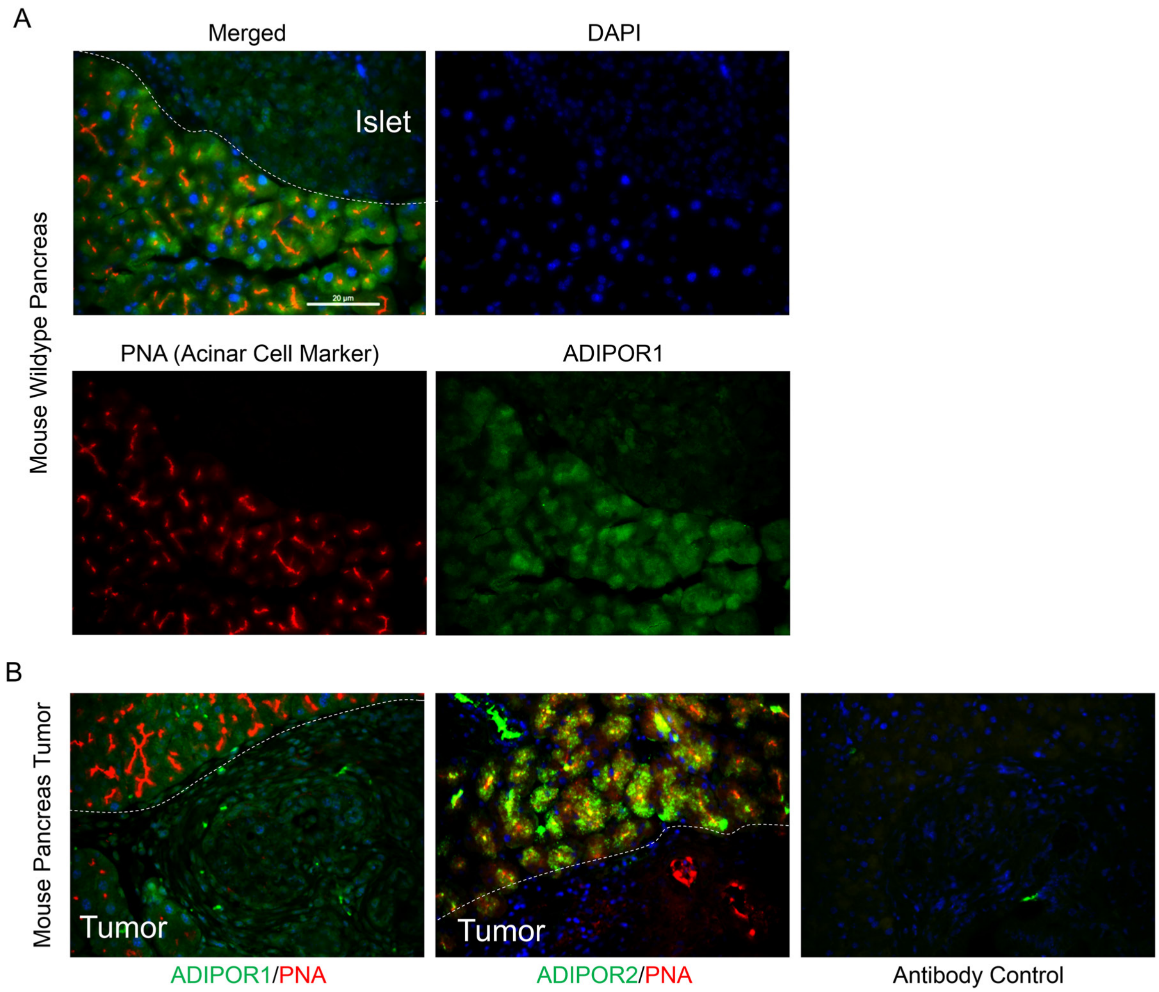

Supplementary Figure 1: ADIPORs are present in pancreatic acinar tissue. (A) Merged and single channel images from pancreatic tissues stained for ADIPOR1 (green) together with Peanut agglutinin (PNA, red) as an acinar cell marker and Dapi (blue) as a nuclear marker. **(A, B)** adiponectin receptor staining was strongest in areas of positive PNA staining, while very low levels of ADIPOR1 and ADIPOR2 staining were seen in the pancreatic islets and tumors, marked by dashed white line. All images were taken at 40x, Scale bar is 20μm.

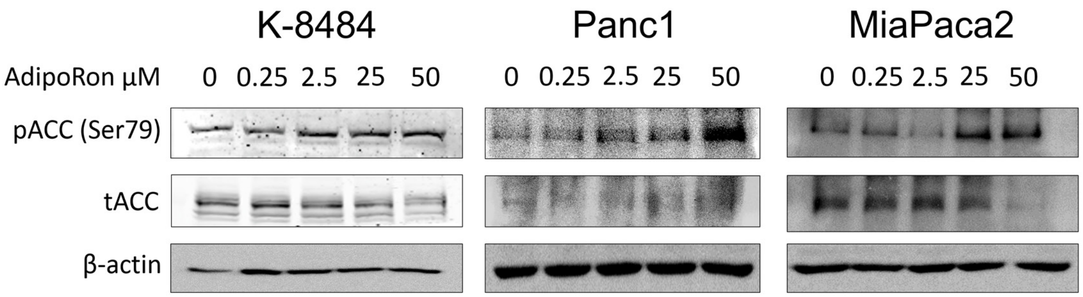

Supplementary Figure 2: AdipoRon increases pACC, a downstream target of AMPK. Mouse K-8484 and human Panc1 and MiaPaca2 PDAC cells were treated with increasing concentrations of AdipoRon for 12h. pACC and tACC (Acetyl-CoA carboxylase) levels were analyzed by western blot analysis, showing greater activation of ACC with higher AdipoRon concentration. β-actin levels are shown as a total loading control.

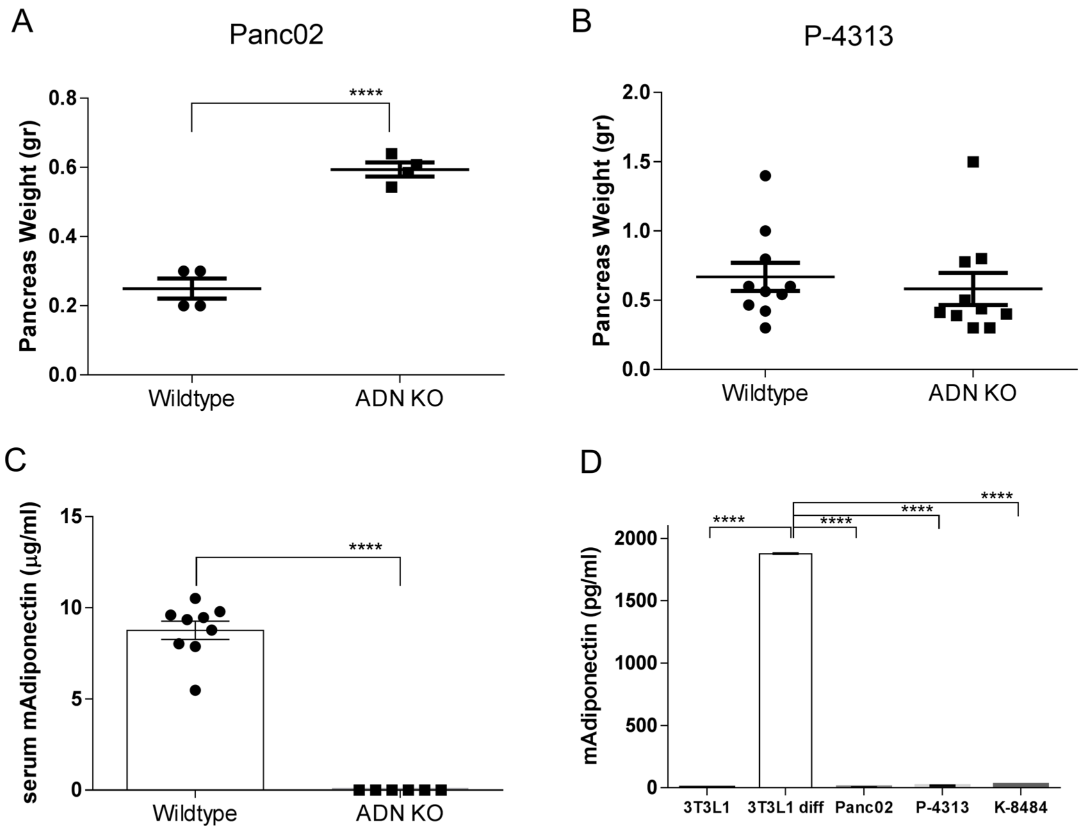

Supplementary Figure 3: Tumor growth in adiponectin deficient mice. Syngeneic pancreatic cancer cells were orthotopically injected into the tail of the pancreas of adiponectin deficient mice (ADN KO). **(A)** growth of Panc02 cells resulted in an increased pancreas-tumor weight in adiponectin deficient mice when compared those in wildtype mice. **(B)** growth of P-4313 cells showed a similar tumor burden when comparing pancreas-tumor weight in wildtype versus ADN KO mice. **(C)** ELISA analysis showed a complete absence of adiponectin in serum from ADN KO mice compared to wildtype mice. **(D)** Conditioned media from murine PDAC cell lines showed undetectable levels of adiponectin compared to conditioned medium from 3T3L1 differentiated adipocytes (3T3L1 diff). Statistical analysis was determined using unpaired t-test (*$P \leq 0.05$, ****$P \leq 0.0001$).
